# Extrakorporale Reanimation – Kriterien, Bedingungen, Outcome: Ein Reality-Check

**DOI:** 10.1007/s00063-022-00913-9

**Published:** 2022-04-11

**Authors:** Ingrid Magnet, Michael Poppe

**Affiliations:** Universitätsklinik für Notfallmedizin, Medizinische Universität Wien, Währinger Gürtel 18–20, 6D, 1090 Wien, Österreich

**Keywords:** Extrakorporale Membranoxygenierung, Herz-Kreislauf-Stillstand, Kardiopulmonale Wiederbelebung, Lungenembolie, Kammerflimmern, Extracorporeal membrane oxygenation, Cardiac arrest, Cardiopulmonary resuscitation, Pulmonary embolism, Ventricular fibrillation

## Abstract

Für ausgewählte Patienten, in denen die konventionelle kardiopulmonale Reanimation (cCPR) erfolglos bleibt, sprechen die europäischen Leitlinien zur Reanimation 2021 erstmals eine Empfehlung zur extrakorporalen Reanimation (eCPR) als mögliche Rettungstherapie aus. Die eCPR wird im therapierefraktären Kreislaufstillstand etabliert, um Diagnostik und Therapie reversibler Ursachen, wie Herzinfarkt, Lungenembolie, akzidentielle Hypothermie, Intoxikationen mit herzwirksamen Substanzen und akute Hypoxie, zu ermöglichen. Selektionskriterien für eCPR umfassen prognostische Reanimationsfaktoren, wie beobachteter Kreislaufstillstand, Start von Reanimationsmaßnahmen in < 5 min, schockbarer Erstrhythmus, Zeichen effektiver cCPR wie Lebenszeichen während der Reanimation, anhaltendes Kammerflimmern, intermittierende Phasen von Spontankreislauf oder anhaltendes endtidales CO_2_ > 10 mm Hg, Patientenalter und Gesundheitszustand. Die Zeitspanne vom Kreislaufstillstand bis zur eCPR ist eine der wichtigsten Determinanten für neurologisch gutes Überleben und sollte < 60 min liegen. Für die Einhaltung dieser Zielvorgabe muss eine entschlossene „Load-and-Go“-Strategie mit frühzeitiger Patientenselektion und raschem Transport unter mechanischer cCPR in ein eCPR-Zentrum verfolgt werden, oder es wird versucht, die eCPR präklinisch zum Einsatz zu bringen. Zwei randomisierte kontrollierte eCPR-Studien demonstrierten Überlebensraten von 43 % bzw. 31,5 % bei Patienten mit anhaltendem Kammerflimmern bzw. kardialem Kreislaufstillstand. Ob diese Ergebnisse außerhalb einzelner hochspezialisierter Zentren anwendbar sind, ist wie die Frage nach der besten präklinischen und innerklinischen Strategie Gegenstand zukünftiger Studien.

## Abstract (en)

In select patients, in whom conventional cardiopulmonary resuscitation (cCPR) fails to re-establish circulation, the 2021 European Resuscitation Council guidelines suggest considering extracorporeal resuscitation (eCPR) as a rescue therapy in settings in which it can be implemented. eCPR is becoming established during refractory cardiac arrest as a bridge-to-therapy for diagnosis and treatment of reversible causes of cardiac arrest, such as myocardial infarction, pulmonary embolism, accidental hypothermia, overdose with cardiotoxic substances, and acute hypoxia. Patient selection criteria comprise prognostic characteristics of cardiac arrest such as witnessed status, resuscitation efforts within 5 min, shockable initial rhythm, and characteristics of effective cCPR such as signs of life during resuscitation, persistent ventricular fibrillation, intermittent episodes of spontaneous circulation or end-tidal CO_2_ levels constantly > 10 mm Hg, patient age and health status. The timeframe from cardiac arrest to eCPR is a major contributor for neurologically favourable survival and should not exceed 60 min according to current guidelines. This may be achieved with an efficient “load & go” strategy, including early patient selection and rapid transport with ongoing mechanical cCPR to the eCPR centre, or with a prehospital eCPR strategy. Two randomized controlled eCPR trials demonstrated survival rates of 43% and 31.5% in patients with refractory ventricular fibrillation and cardiac-origin cardiac arrest, respectively. Whether these results are generalizable outside such highly specialized centres, and which prehospital and in-hospital strategy is best for which patients, remains to be determined in future studies.

## Hintergrund und Rationale

Sofortige und qualitativ hochwertige Herzdruckmassage ohne Unterbrechungen sowie frühzeitige Defibrillation sind die wichtigsten Maßnahmen für neurologisch gutes Überleben nach Herz-Kreislauf-Stillstand (HKS). Für ausgewählte Patienten, in denen die konventionelle kardiopulmonale Reanimation (cCPR) erfolglos bleibt, wird in den europäischen Leitlinien zur Reanimation aus dem Jahr 2021 die extrakorporale Reanimation (eCPR) erstmals als mögliche Rettungstherapie angeführt [1].

Die eCPR ist eine venoarterielle extrakorporale Membranoxygenierung

Die eCPR ist eine venoarterielle extrakorporale Membranoxygenierung (va-ECMO), die im therapierefraktären Kreislaufstillstand etabliert wird, um Kreislauf und Gasaustausch zu ersetzen [2]. Für eine va-ECMO wird Blut über eine Kanüle aus dem rechten Vorhof sowie dem Hohlvenensystem drainiert, durch eine Lungenmembran mit Gasaustauscher gepumpt und über eine Kanüle in ein zentrales arterielles Gefäß zurückführt. In der eCPR erfolgen Punktion und Anlage der benötigten großlumigen Kanülen unter fortgesetzter Herzdruckmassage und nahezu immer über die Vena femoralis sowie Arteria femoralis communis. Die Versorgung mit oxygeniertem Blut erfolgt retrograd über die Aorta bis in die zerebrale und koronare Strombahn [3].

Während die cCPR ein Herzzeitvolumen (HZV) < 25 % mit entsprechend herabgesetzter zerebraler und koronarer Perfusion produziert, kann die va-ECMO nahezu das gesamte HZV übernehmen und über Tage bis Wochen aufrechterhalten. Zweck der eCPR ist, die Durchblutung lebenswichtiger Organe, insbesondere Gehirn und Herz, so lange zu gewährleisten, bis ein Spontankreislauf („return of spontaneous circulation“, ROSC) wiedererlangt wird. Die eCPR ist somit nicht Therapieziel, sondern Überbrückungsmaßnahme zur Diagnostik und Behandlung reversibler HKS-Ursachen („bridge to therapy“) oder um dem Körper Zeit zur Genesung zu geben („bridge to recovery“). Die Wahrscheinlichkeit für neurologisch gutes Überleben (Cerebral Performance Category [CPC] 1–2 oder Modified Rankin Scale [mRS] 0–3) nimmt mit Dauer der Reanimation drastisch ab und liegt nach 30-minütiger CPR bei < 5 %. Die eCPR kann bei gut funktionierender Rettungskette diese Zeitspanne deutlich verlängern und neurologisch gutes Überleben auch nach > 60 min Reanimation in bis zu 19 % der Patienten ermöglichen [4].

Die eCPR kann neurologisch gutes Überleben auch nach > 60 min Reanimation ermöglichen

Der von der Extracorporal Life Support Organisation (ELSO) aktuell gebräuchliche Begriff eCPR für eine va-ECMO während laufender Reanimation löst in dieser Publikation andere Bezeichnungen dieser Anwendung wie ECMO-CPR, Emergency Cardiopulmonary Bypass (ECPB) oder Extrakorporal Life Support (ECLS) ab [2].

## Indikation gemäß Leitlinienempfehlungen

In Tab. 1 sind rezente und erwähnenswerte Studien zum Therapieerfolg von eCPR zusammengefasst. Das International Liaison Committee on Resuscitation (ILCOR) und das European Resuscitation Council (ERC) haben die eCPR auf Basis dieser Evidenzlage in ihre aktuellen Leitlinien als mögliche Rettungstherapie für ausgewählte Patienten mit Kreislaufstillstand innerhalb und außerhalb des Krankenhauses („in-hospital cardiac arrest“ [IHCA], „out-of-hospital cardiac arrest“ [OHCA]) aufgenommen, wenn eine cCPR erfolglos bleibt und eine behandelbare Ursache des Kreislaufstillstands vermutet wird [1, 2, 5, 6]. Mögliche Indikationen zur eCPR sind gemäß aktueller Leitlinien

- die perkutane Koronarintervention und Erholung von myokardialem „stunning“ bei Patienten mit akutem Herzinfarkt,
- die Thrombolyse oder Thrombektomie bei massiver Lungenembolie,
- die Wiedererwärmung bei akzidentieller Hypothermie,
- der Abbau herzwirksamer Substanzen bei akut lebensbedrohlichen Vergiftungen oder Hyperkaliämie sowie
- Hypoxie und Lungenversagen z. B. bei Ertrinkungsopfern oder Asthmakrise.

**Table 1:** 

Studien	Autor (Publikationsjahr)	Land/Region (Beobachtungszeitraum)	Untersuchte Patienten	Outcomeparameter	Resultate bzw. Interventionen
*Abgeschlossene Studien*					
Nichtrandomisierte Studien und Kohortenanalysen	Chen (2008) [10]	Taiwan (2004–2006)	IHCA: 46 Propensity-Score-gemachte Paare eCPR und cCPR	Überleben bei Entlassung aus Krankenhaus	eCPR vs. cCPR: 30,4 % vs. 15,2 %, (*p* < 0,0001)
	Bourcier (2022) [9]	Frankreich (2007–2019)	IHCA: 137 eCPR	Überleben bei Entlassung aus ICU, Überleben zu 1 Jahr	21,9 % Überleben ICU, 19 % Überleben 1 Jahr
	Lunz (2020) [12]	Deutschland, Italien, Schweden (2012–2016)	IHCA: 165 eCPR OHCA: 258 eCPR	CPC 1–2 nach 3 Monaten	IHCA: 36 %; OHCA: 9 %
	Lamhaut (2017) [11]	Frankreich (2007–2019)	OHCA; P1: 114 eCPR (46 präklinisch), P2: 42 eCPR (27 präklinisch)	CPC 1–2 bei Entlassung aus ICU oder nach 1 Monat	Periode 1 vs. Periode 2: 8 % vs. 29 % (*p* < 0,001)
	Bartos (2020) [4]	USA (2015–2019)	OHCA: 133 eCPR, 654 cCPR	Überleben bei Entlassung aus Krankenhaus	eCPR vs. cCPR: 39 % vs. 23 % (*p* < 0,001)
Randomisierte Studien	Yannopoulos (2020) [7]	USA (2019–2020)	OHCA: 15 eCPR, 15 cCPR	Überleben bei Entlassung aus Krankenhaus	eCPR vs. cCPR: 43 % vs. 7 % („risk difference“ 36 %, 95 %-Konfidenzintervall 3,7–59,2)
	Belohlavek (2022) [8]	Tschechien (2013–2021)	OHCA: 124 eCPR, 132 cCPR	CPC 1–2 nach 6 Monaten	eCPR vs. cCPR: 31,5 % vs. 22,0 %, (*p* = 0,09)
Metaanalysen	Gravesteijn (2020) [14]	International (2002–2016)	IHCA: 19 Studien, 1011 eCPR	Überleben bei Entlassung aus Krankenhaus, CPC 1–2 bei Entlassung aus Krankenhaus	30 % Überleben, 25 % CPC 1–2
	Downing (2022) [13]	International (2000–2020)	OHCA: 44 Studien, 3097 eCPR	Überleben bei Entlassung aus Krankenhaus, CPC 1–2 bei Entlassung aus Krankenhaus	24 % Überleben, 18 % CPC 1–2
	Scquizzato (2022) [15]	Südkorea, Tschechien, Frankreich, Japan, USA (2000–2021)	OHCA: 6 Studien, 584 eCPR, 593 cCPR	CPC 1–2 zum spätesten Studienzeitpunkt	eCPR vs. cCPR: 14 % vs. 7,8 % (*p* < 0,001)
*Laufende Studien*					
–	INCEPTION Trial (NCT03101787)	Niederlande	110 Patienten	CPC 1–2 nach 1 Monat	1:1-Randomisierung innerklinische eCPR vs. cCPR bei OHCA
–	ON-SCENE Trial (NCT04620070)	Niederlande	390 Patienten	Überleben bei Entlassung aus Krankenhaus; CPC 1–2 nach 6 und 12 Monaten	OHCA mit präklinischer eCPR, Stepped-wedge-Design
–	APACAR2 Trial (NCT02527031)	Frankreich	210 Patienten	CPC 1–2 nach 6 Monaten	Innerklinische vs. präklinische eCPR bei OHCA
Machbarkeitsstudie	SUB30 Trial (NCT03700125)	London	6 Patienten	Präklinische eCPR binnen 30 min nach Kreislaufstillstand	–

*eCPR* extrakorporale kardiopulmonale Reanimation, *cCPR* konventionelle kardiopulmonale Reanimiation, *IHCA *„in-hospital cardiac arrest“, *OHCA* „out-of-hospital cardiac arrest“, *CPC* „cerebral performance category“, *OR* Odds-Ratio

## Evidenz und Outcome

### Kardialer Kreislaufstillstand

Therapierefraktäres Kammerflimmern, zumeist definiert als ≥ 3 nichterfolgreiche Defibrillationen, oder vermutete kardiale Genese sind in den Selektionskriterien der meisten eCPR-Studien enthalten (siehe Tab. 1). Eine erste randomisierte kontrollierte Studie von Yannopoulos et al. zeigte bereits in der ersten Interimsanalyse nach insgesamt 30 Patienten mit therapierefraktärem Kammerflimmern deutlich verbessertes neurologisches Überleben für eCPR gegenüber cCPR (43 % vs. 7 %) und wurde frühzeitig beendet [7].

In einer zweiten randomisierten kontrollierte Studie von Bělohlávek et al. an Patienten mit OHCA und vermuteter kardialer Genese zeigte eCPR im Vergleich mit cCPR einen Trend zu besserem neurologischem Überleben, jedoch ohne statistisch signifikanten Unterschied (31,5 % vs. 22,0 %; *p* = 0,09; [8]). Im Unterschied zu Yannopoulos et al. hatten nur 58 % der Patienten Kammerflimmern als Erstrhythmus und Patienten wurden bereits präklinisch und nicht erst bei Eintreffen im Krankenaus in den jeweiligen Studienarm randomisiert, was die verschiedenen Überlebensraten erklären könnte. In der Subgruppe ≥ 45 min Reanimation hatten 24 von 26 Patienten mit neurologisch gutem Überleben eine eCPR erhalten. Darüber hinaus weisen zahlreiche nichtrandomisierte Studien und Kohortenanalysen [4, 9–12] sowie Metaanalysen [13–15] auf verbessertes Überleben mit eCPR in dieser Patientengruppe hin.

### Lungenembolie

In einer Metaanalyse zu eCPR ohne cCPR-Kontrollgruppe bei Kreislaufstillstand durch Lungenembolie überlebten 65 % (64 von 99) der Patienten, 88 % davon mit neurologisch gutem Überleben, wobei in der Literatur eine Mortalität von bis zu 90 % angegeben wird [16]. Eine Registerstudie zu Lungenembolie und Kreislaufstillstand zeigte einen Überlebensvorteil für den sehr kleinen Anteil der Patienten mit eCPR und Thrombolyse, eCPR und Thrombektomie, aber auch eCPR allein ohne Reperfusionstrategie gegenüber der Behandlung mit Thrombolyse allein [17]. Eine ELSO-Registerstudie der Jahre 1997–2017 fand in 42 schwangeren Patienten mit eCPR eine Überlebensrate von 55 % [18]. Die eCPR-Ursachen umfassten peri- und postpartale Kardiomyopathie sowie Lungenembolie inklusive Fruchtwasserembolie.

### Akzidentielle Hypothermie

In rezenten Metaanalysen von Patienten mit akzidentieller Hypothermie und eCPR lag das Überleben bei 73 % (153 von 210 Patienten) für beobachteten und 27 % (60 von 221 Patienten) für nichtbeobachteten Kreislaufstillstand mit jeweils neurologisch gutem Überleben von 89 % respektive 83 % [19, 20]. Hypotherme Patienten mit hämodynamischer Instabilität sollten daher a priori an Zentren mit Möglichkeit zur eCPR transferiert werden. Für die Indikationsstellung zur eCPR können Prädiktoren wie z. B. der HOPE-Score herangezogen werden [21]. Der EKG-Erstrhythmus fließt in dieses Prognosetool nicht ein. So fand sich bei 48 % (28 von 60) der Überleber eines unbeobachteten hypothermieassoziierten Kreislaufstillstands eine Asystolie als Erstrhythmus, 79 % davon zeigten neurologisch gutes Überleben [20].

## Patientenselektion

Robuste Selektionskriterien sollen jene Patienten mit dem größten Nutzen und der größten Überlebenswahrscheinlichkeit durch eCPR finden. Die Tab. 2 zeigt häufige Einschlusskriterien in eCPR-Studien, deren prospektive Evaluierung steht allerdings noch aus. Der Anteil der potenziellen eCPR-Patienten im HKS-Gesamtkollektiv wird zwischen 6 und 11 % angegeben [8, 22]. Durch die Seltenheit wird der frühzeitigen und akkuraten Identifikation dieser Patienten auf allen Ebenen der Versorgung, beginnend bei der Rettungsleitstelle über den Rettungsdienst bis zur aufnehmenden Klinik, eine tragende Rolle zuteil.

**Table 2:** 

Item	ELSO 2021 [2]	Belohlavek 2022 [8]	Yannopoulos 2020 [7], Bartos 2020 [4]	INCEPTION Trial (NCT03101787)	Lamhaut 2017 Periode 2 [11]
Zeit bis Strategiewechsel	HKS seit 10–15 min	HKS >5 min	Kein ROSC nach 3 Schocks	HKS ≥15 min	HKS >20 min
Alter	< 70	18–65	18–75	18–70	–
Lebenszeichen	Positiver Marker für Überleben	–	–	–	Lebenszeichen unter Reanimation
Beobachteter Kollaps	Beobachteter Kollaps	Beobachteter Kollaps	–	Beobachteter Kollaps	Beobachteter Kollaps
Ersthelferreanimation	Weniger als 5 min „no-flow“	–	–	Durchgeführte Ersthelferreanimation	Weniger als 5 min „no-flow“
Erstrhythmus/HKS-Genese	Alle Rhythmen außer Asystolie	Alle Rhythmen/kardiale HKS-Genese	Schockbar	Schockbar	Alle Rhythmen außer Asystolie
Intermittierende ROSC-Phasen	Intermittierende ROSC-Phasen oder anhaltendes Kammerflimmern	–	–	–	–
Endtidales CO_2_	> 10 mm Hg während cCPR	–	> 10 mm Hg während cCPR	–	> 10 mm Hg während cCPR
Zeitrahmen und System	cCPR < 60 min	cCPR bis Eintreffen im Krankenhaus < 60 min	Transportzeit < 30 min, LUCAS verfügbar	cCPR bis Kanülierung < 60 min	cCPR bis Kanülierung < 100 min (Ziel: < 60 min)
Komorbidität als Kontraindikation	Terminale Erkrankung (z. B. Herzinsuffizienz, COPD, Leber‑, Nierenversagen), Patientenwusch	CPC ≥ 3	Terminale Erkrankung	Herzinsuffizienz NYHA ≥ 3 COPD Gold ≥ 3 CPC ≥ 3	Schwere Vorerkrankungen
Komplikationen als Kontraindikation	Höhergradige Aortenklappeninsuffizienz	Blutungsneigung, Verdacht auf Insult oder intrakranielle Blutung, Schwangerschaft	Ausgeprägte Blutungen	Polytrauma, femorale Bypasoperation, Schwangerschaft	–

*cCPR* konventionelle kardiopulmonale Reanimiation, *eCPR* extrakorporale kardiopulmonale Reanimation, *CPC* „cerebral performance category“, *ELSO* Extracorporeal Life Support Organization,* HKS* Herz-Kreislauf-Stillstand, *IHCA* „in-hospital cardiac arrest“, *OHCA* „out-of-hospital cardiac arrest“, *ROSC* „return of spontaneous circulation“, *NYHA* Graduierung der Herzinsuffizienz gemäß New York Heart Association in 1–4 , *COPD* „chronic obstructive pulmonary disease“, *LUCAS* Thoraxkompressionssystem, Medtronic, Tolochenaz, Schweiz

### Patientencharakteristika und Umstände des Kreislaufstillstands

Junges Patientenalter und damit niedrige Rate an Komorbiditäten, [10] beobachteter Kollaps, frühzeitige (Laien‑)Reanimation und das Vorhandensein einer reversiblen (z. B. kardialen) HKS-Ursache sind Prädiktoren für neurologisch gutes Überleben und in vielen Studien absolute Selektionskriterien für eCPR. Das Überleben von eCPR-Patienten mit schockbarem Erstrhythmus ist signifikant höher als ohne, jedoch konnte auch bei nichtschockbaren Erstrhythmen eine hohe Überlebensrate erzielt werden, wenn die Zeit bis zur eCPR sehr kurz blieb [12]. Ein Kreislaufstillstand im öffentlichen Raum ist ebenso mit deutlich höheren Überlebensraten assoziiert, vermutlich da er öfter beobachtet ist, mehr schockbare Rhythmen vorliegen und die Reanimation und Defibrillation früher stattfinden [23].

### Zeichen einer effektiven Perfusion unter Reanimation

Qualitativ hochwertige cCPR ist für eine erfolgreiche eCPR unerlässlich. Lebenszeichen, wie Pupillenreaktion, Schnappatmung oder Anzeichen von Bewusstsein während der Reanimation („signs of life“), erhöhen die Chance auf neurologisch gutes Überleben [24]. Das endtidale CO_2_ (etCO_2_) ist ein bewährter Parameter für Reanimationsqualität, anhaltend niedriges etCO_2_ (< 10 mm Hg) wird als Prädiktor für Tod und damit als Ausschlussgrund für eCPR herangezogen [7]. Anhaltend niedrige Werte der regionalen zerebraler Sauerstoffsättigung (rSO_2_) korrelieren ebenso mit Patienten ohne Überleben und könnten einen weiteren Baustein zur Indikationsstellung bzw. zum Abbruch einer eCPR Therapie darstellen [25]. Metabolische Parameter, wie niedriges Serumlaktat und höherer pH-Wert zum Zeitpunkt der eCPR-Implantation, korrelieren mit dem Überleben, es konnten sich aber keine klar definierten Grenzwerte zum Ausschluss einer eCPR etablieren [4].

### Systemseitige Kriterien

Die systemseitigen Voraussetzungen für eCPR sind beträchtlich und umfassen Equipment, wie eine mechanische Reanimationshilfe oder vorbereitete eCPR-Sets, sowie Personal, das in der Implantation und Betreuung einer eCPR geschult und erfahren ist. In Zentren mit < 30 ECMO-Anwendungen pro Jahr nimmt die Mortalität signifikant zu [26]. Eine entscheidende systemseitig beeinflussbare Determinante für neurologisch gutes Überleben ist die Zeitspanne von Kreislaufstillstand bis Start der eCPR, die nur durch enge Zusammenarbeit der handelnden Organisationen (Präklinik, Klinik, eCPR-Team) minimiert werden kann [4, 27]. Weitere wesentliche Aufgaben eines eCPR-Programms sind die rasche Diagnostik und Behandlung reversibler HKS-Ursache, Postreanimationsbehandlung nach aktuellen Leitlinien inklusive Targeted Temperature Management (TTM) sowie Erkennen und Beherrschen von Komplikationen [2, 6].

### Innerklinischer Kreislaufstillstand

Die ersten Studien mit Vorteil von eCPR gegenüber cCPR wurden bei IHCA durchgeführt [10]. Seitdem konnten Kohortenstudien und Metaanalysen eine stabile Überlebensrate für eCPR bei IHCA von 30 % zeigen, [14] randomisierte kontrollierte Studie sind bis heute nicht abgeschlossen. Patienten mit IHCA sind öfter beobachtet, die Zeit bis zur Reanimation und Defibrillation sowie zum Start der eCPR ist kürzer und die Überlebensraten besser [12]. Selektionskriterien für eCPR im IHCA bzw. OHCA können sich somit unterscheiden.

Die Selektionskriterien für eine eCPR im IHCA bzw. OHCA können sich unterscheiden

Im Rahmen der Etablierung eines Scores (RESCUE-IHCA) zur Mortalitätsprognose bei Patienten mit IHCA und eCPR waren Patientenalter, Uhrzeit des Kreislaufstillstands, Erstrhythmus, vorbekannte Niereninsuffizienz, Krankheitsgeschichte (internistisch oder chirurgisch, kardiologisch oder nichtkardiologisch) sowie Reanimationsdauer mit der Krankenhausmortalität assoziiert [28]. Ebenso konnte ein Algorithmus bestehend aus 3 Kriterien (Alter, Erstrhythmus und Dauer der konventionellen Reanimation) die Mortalität auf der Intensivstation von IHCA-Patienten nach eCPR voraussagen und damit die Patientenselektion verbessern [9].

## Zeitoptimierung und präklinisches Management

**Figure 1:**
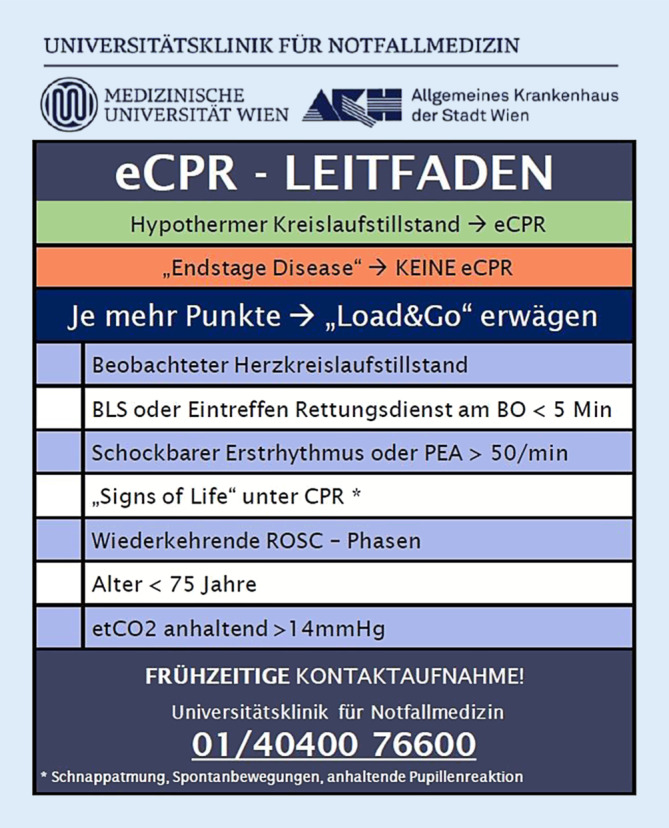

Der optimale Zeitpunkt zum Start der eCPR ist nicht geklärt, wird für OHCA aber mit einer 30-minütigen Reanimationsdauer angenommen. Jede Verzögerung der eCPR um weitere 10 min verringert das neurologisch gute Überleben um 25 % [4]. Die Empfehlung mehrerer Fachgesellschaften lautet, eCPR innerhalb von 60 min nach HKS zu etablieren [2, 4, 6]. Internationale Studien berichten eine Zeitspanne für HKS bis eCPR von 45–53 min bei IHCA [9, 10] und 58–67 min bei OHCA, wobei die Implantationszeit mit 12–15 min angegeben wird und der Transport des Patienten zur eCPR den größten Verzögerungsfaktor darstellt [7, 8, 12, 27]. Um diese Zeitvorgabe zu erreichen, muss eine entschlossen „Load-and-Go“-Strategie mit raschem Transport unter mechanischer cCPR in das eCPR-Zentrum verfolgt werden. Das eCPR-Team sollte frühzeitig aktiviert werden, sobald ein Patient als potenzieller eCPR-Kandidat erkannt wird, was in einigen eCPR-Studien [7] und auch im eCPR-Programm der Autoren nach 3 Rhythmusanalysen geschieht (Abb. 1), möglicherweise aber bereits zum Zeitpunkt des Notrufs erfolgen sollte [8]. Eine Machbarkeitsstudie versuchte die Zeit von Notruf bis Ankunft im Krankenhaus auf ≤ 30 min zu verkürzen, konnte dieses ehrgeizige Ziel aber nur in 5 von 12 Patienten erreichen [29].

Es muss eine „Load-and-Go“-Strategie mit raschem Transport unter mechanischer cCPR verfolgt werden

Eine alternative Strategie zur Verkürzung dieser Zeitspanne ist die Verlegung der eCPR aus der Klinik zum Patienten – die präklinische eCPR. Der potenzielle Nutzen durch Zeitersparnis und die Vermeidung von Transport-CPR muss allerdings den potenziellen Nachteilen, wie erschwertes Personal‑, Material- und Komplikationsmanagement (z. B. Blutungen, Infektionen), gegenübergestellt werden. Die wenigen publizierten Daten berichteten eine Dauer von 87 ± 27 min vom HKS bis zum Start eCPR, [11], länger als bei innerklinischer eCPR-Implantation [7, 8]. Weitere Studien, die das Konzept präklinischer eCPR erforschen, sind in der Planung/Durchführung (ON-SCENE Trial, NCT04620070; APACAR2 Trial, NCT02527031). Ein besonders ambitioniertes Projekt ist die SUB30-Studie (NCT03700125). Hier soll eine eCPR möglichst in weniger als 30 min nach Kollaps erfolgreich etabliert werden. Ein weiteres Konzept verfolgt den Transport von eCPR-Patienten in eines von 3 strategisch gut gelegenen Krankenhäusern, wo sie auf ein mobiles eCPR-Team treffen, wodurch Vorteile präklinischer und innerklinischer eCPR verbunden werden sollen. Erst nach Etablierung der eCPR und Durchführung der Koronarangiographie werden die Patienten in das eCPR-Zentrum verlegt [30].

## Wiener Modell

Das eCPR-Programm am Reanimationszentrum der Medizinischen Universität Wien besteht seit dem Jahr 1993, im letzten Jahrzehnt erfolgten circa 30 eCPR-Anwendungen für OHCA und IHCA pro Jahr. In enger Zusammenarbeit zwischen Rettungsdienst und Reanimationszentrum wurden gemeinsame Standardarbeitsanweisungen (SOP) zur eCPR-Versorgung erarbeitet. Alle Sanitäter und Notärzte sind mit einem Leitfaden für eCPR ausgestattet (Abb. 1), der die frühzeitige präklinische Weichenstellung zur eCPR ermöglichen soll. Besonderer Bedeutung kommt dem „Field Supervisor“ zu, einem Notfallsanitäter, der zur Qualitätskontrolle sowie organisatorischen Unterstützung zusätzlich zum Einsatzort disponiert wird und die Kommunikation zwischen Präklinik und Reanimationszentrum übernimmt. Noch vor Eintreffen des Patienten werden Charakteristika des Patienten und Umstände des Kreislaufstillstands im Telefonat mit dem Ersthelfer und dem Rettungsdienst sowie aus der elektronischen Krankengeschichte erfasst.

Die eCPR wird im Akutbehandlungsbereich der Klinik für Notfallmedizin in Zusammenarbeit mit der Herzchirurgie (eCPR-Team) etabliert. Nach Übernahme des Patienten durch das Advanced-life-support(ALS)-Team der Klinik für Notfallmedizin erfolgt die Überprüfung der ALS-Maßnahmen inklusive orientierender Ultraschalluntersuchung und schließlich ein zusammenfassendes Team-Time-Out mit Entscheidung für oder gegen eCPR. Bereits währenddessen punktiert das eCPR-Team nach Gabe von 5000 I.E. Heparin perkutan die großen Femoralgefäße und bringt Führungsdrähte ein. Bei Entscheidung für eCPR wird durch das ALS-Team per transösophagealer Echokardiographie die Lage der Führungsdrähte kontrolliert und die Herzdruckmassagequalität überwacht. Mit Start der eCPR erfolgt, bei fehlenden Kontraindikationen, ein „targeted temperature management“ (TTM) auf 33 °C durch den extrakorporalen Kreislauf, die Anlage einer distalen antegraden Beinkanüle sowie die Einleitung einer empirischen antimikrobiellen Therapie. Bei Verdacht auf eine primär kardiale HKS-Genese erfolgt unmittelbar die Koronarintervention. Andernfalls oder nach der Intervention wird eine standardisierte Computertomographie (CT) durchgeführt, bestehend aus kranialer CT, CT-Angiographie der gesamten Aorta bis zur Einmündung der antegraden Beinkanüle sowie CT von Thorax und Abdomen.

Noch vor Eintreffen des Patienten werden die Umstände des Kreislaufstillstands erfasst

Das eCPR-Programm wurde während der Pandemie durch die Coronaviruserkrankung 2019 (COVID-19) unter Berücksichtigung der persönlichen Sicherheit aller Mitarbeiter fortgeführt. Zwei von 34 eCPR-Patienten im Jahr 2020 wurden COVID-19-positiv getestet, in einem Fall wurde die HKS-Ursache als primär kardial, im zweiten als Lungenembolie gewertet.

## Ausblick und offene Fragen

Erste randomisierte kontrollierte Studien konnten bei ausgewählten Patienten einen Überlebensvorteil für ein Therapiekonzept mit eCPR, hochwertiger cCPR mit frühzeitigem Transport in ein eCPR-Zentrum und standardisierter Postreanimationsbehandlung inklusive unmittelbarer Herzkatheteruntersuchung und TTM zeigen. Die Generalisierbarkeit dieser Ergebnisse außerhalb einzelner hochspezialisierter Zentren wird in multizentrischen Studien geprüft werden müssen. Darüber hinaus bleiben offene Fragen hinsichtlich der Patientenselektion und vor allem hinsichtlich der Optimierung der eCPR-Logistik im Rettungsdienst sowie in der Klinik. Die beste präklinische und innerklinische eCPR-Strategie muss erst gefunden werden und wird sich wahrscheinlich je nach Rettungs- und Krankenhauslandschaft sowie Bevölkerungsverteilung unterscheiden.

## Fazit für die Praxis

- Für ausgewählte Patienten, in denen die konventionelle kardiopulmonale Reanimation (cCPR) erfolglos bleibt und eine behandelbare Ursache des Kreislaufstillstands vermutet wird, sprechen die europäischen Leitlinien zur Reanimation im Jahr 2021 erstmals eine Empfehlung für extrakorporale Reanimation (eCPR) als mögliche Rettungstherapie aus.
- Mögliche Indikationen zur eCPR sind Herzinfarkt, Lungenembolie, akzidentielle Hypothermie, Intoxikation herzwirksamer Substanzen und akute Hypoxie.
- Die Patientenselektionskriterien umfassen beobachteter Kreislaufstillstand, Start der Reanimation < 5 min, schockbarer Erstrhythmus, Lebenszeichen unter Reanimation, anhaltendes Kammerflimmern, intermittierender Spontankreislauf, endtidales CO_2_ anhaltend > 10 mm Hg, Patientenalter und Gesundheitszustand.
- Für neurologisch gutes Überleben sollten zwischen Kreislaufstillstand und eCPR < 60 min liegen. Dazu muss das eCPR-Team aktiviert werden, sobald ein Patient als potenzieller eCPR-Kandidat erkannt wird.
